# Compact and Interpretable Neural Networks Using Lehmer Activation Units

**DOI:** 10.3390/e28020157

**Published:** 2026-01-31

**Authors:** Masoud Ataei, Sepideh Forouzi, Xiaogang Wang

**Affiliations:** 1 Department of Mathematical and Computational Sciences, University of Toronto, Mississauga, ON L5L 1C6, Canada; 2 Department of Computer Science, University of Colorado Boulder, Boulder, CO 80309, USA; 3 Department of Mathematics and Statistics, York University, Toronto, ON M3J 1P3, Canada

**Keywords:** Lehmer transform, interpretable neural networks, complex-valued learning, nonlinear activation functions, deep learning, universal approximation theorem

## Abstract

We introduce Lehmer Activation Units (LAUs), a class of aggregation-based neural activations derived from the Lehmer transform that unify feature weighting and nonlinearity within a single differentiable operator. Unlike conventional pointwise activations, LAUs operate on collections of features and adapt their aggregation behavior through learnable parameters, yielding intrinsically interpretable representations. We develop both real-valued and complex-valued formulations, with the complex extension enabling phase-sensitive interactions and enhanced expressive capacity. We establish a universal approximation theorem for LAU-based networks, providing formal guarantees of expressive completeness. Empirically, we show that LAUs enable highly compact architectures to achieve strong predictive performance under tightly controlled experimental settings, demonstrating that expressive power can be concentrated within individual neurons rather than architectural depth. These results position LAUs as a principled, interpretable, and efficient alternative to conventional activation functions.

## 1. Introduction

Recent advances in deep learning have enabled remarkable performance across a wide range of tasks, from computer vision to scientific data analysis. These gains, however, have largely been driven by increasingly deep and overparameterized architectures, often involving millions or billions of trainable parameters. While such models are highly expressive, their reliance on depth and scale comes at the cost of substantial computational overhead and limited interpretability. The resulting black-box behavior poses challenges not only for deployment in resource-constrained environments, but also for understanding, verifying, and trusting model predictions in high-stakes applications.

These limitations have motivated growing interest in neural architectures that prioritize efficiency and interpretability alongside predictive accuracy. A central question is whether complex nonlinear relationships can be modeled using compact architectures whose internal mechanisms remain transparent and mathematically well understood. Addressing this question requires rethinking one of the most fundamental components of neural networks: the activation function. Conventional activations are typically applied elementwise and are fixed a priori, placing the burden of expressivity primarily on network depth. In contrast, aggregation-based nonlinearities offer the possibility of capturing higher-order feature interactions within individual units, potentially reducing architectural complexity while improving interpretability.

In this work, we propose a new class of activation functions based on the Lehmer transform, a parameterized family of means with well-established mathematical structure [1]. The Lehmer transform provides a continuous interpolation between min-like and max-like aggregation behaviors via a single order parameter, and naturally admits a weighted extension that enables adaptive feature attribution through trainable importance coefficients. This yields an explicit and interpretable nonlinear mechanism: each unit learns both which coordinates matter (via weights) and how they should be aggregated (via the Lehmer order), in a fully differentiable and structurally transparent form.

Building on these properties, we introduce Lehmer Activation Units (LAUs) and develop both real-valued and complex-valued formulations. Real-valued LAUs implement adaptive, learnable aggregation within individual neurons, enabling a continuous transition between suppressive and amplifying regimes governed by the learned Lehmer order. We further extend this construction to the complex domain by allowing the Lehmer order to be complex-valued, introducing oscillatory modulation through complex exponentiation. This extension enables phase-sensitive interactions and richer relational modeling while remaining compatible with gradient-based optimization and modern training pipelines, in the spirit of complex-valued neural networks developed for structured and phase-bearing data.

The proposed activation functions give rise to compact and interpretable neural architectures, where compactness refers to architectural and representational efficiency through fewer units and parameters. For tabular data, we evaluate deliberately minimal networks in which a single dense layer with only a single LAU is used to isolate the expressive contribution of the activation itself. For image classification, we integrate LAUs directly into convolutional networks, employing Lehmer-based activations within convolutional feature extraction and within the classification head, together with standard normalization and pooling operators to ensure stable training. Across these settings, the resulting models aim to achieve competitive accuracy with substantially reduced architectural complexity and improved transparency.

The contributions of this work are threefold. First, we introduce LAUs as a mathematically grounded alternative to conventional pointwise activations, incorporating the aggregation structure of the Lehmer transform and establishing a universal approximation theorem for LAU-based networks. Second, we develop both real-valued and complex-valued variants, enabling magnitude-driven aggregation and phase-sensitive modulation within compact models. Third, we demonstrate empirically that LAU-based architectures achieve strong performance on standard tabular and image benchmarks while substantially reducing network depth and parameter count.

The remainder of this paper is organized as follows. Section 2 provides an overview of relevant literature and prior modeling approaches. Section 3 establishes the mathematical framework of the Lehmer transform, including its weighted and complex-valued extensions. Section 4 introduces LAUs and discusses their integration into neural network architectures. Section 5 presents the experimental setup and computational results, highlighting the performance and efficiency of LAU-based models. Section 6 concludes the paper and outlines directions for future research.

## 2. Literature Review

The rapid growth in model complexity and parameterization in modern deep learning has intensified concerns regarding interpretability, transparency, and trustworthiness. While large-scale neural networks have achieved remarkable empirical success, their opaque decision-making processes pose challenges in domains where accountability and human understanding are essential, such as healthcare, finance, and scientific discovery [2,3,4]. This has motivated extensive research into interpretable machine learning methods that aim to provide insight into model behavior without sacrificing predictive performance.

A prominent line of work focuses on post-hoc interpretability techniques, which seek to explain model predictions after training. Methods such as saliency maps [5], Layer-wise Relevance Propagation (LRP) [6], and Local Interpretable Model-agnostic Explanations (LIME) [7] analyze feature relevance by probing trained models. Although these approaches offer intuitive explanations, they do not alter the underlying model structure and therefore provide limited guarantees regarding interpretability during training or robustness under distributional shifts.

To address these limitations, recent research has emphasized intrinsic interpretability, embedding explanatory mechanisms directly into neural architectures. Attention mechanisms [8,9] provide soft, data-dependent weighting of features or tokens and have become a standard component in sequence modeling and vision architectures. Self-explaining neural networks [10] and concept-based approaches [11] further promote transparency by explicitly associating predictions with human-interpretable concepts. Graph Neural Networks (GNNs) [12] also offer a degree of structural interpretability by operating on relational data with explicit node and edge semantics. Despite these advances, many intrinsically interpretable models still rely on deep architectures and pointwise nonlinearities, leaving open the question of whether interpretability and expressivity can be achieved within significantly more compact models.

In parallel, complex-valued neural networks (CVNNs) have emerged as a powerful extension of real-valued architectures, particularly for data exhibiting oscillatory, periodic, or phase-sensitive structure. By representing activations and parameters in the complex plane, CVNNs are able to encode relationships that are difficult to capture using purely real-valued models [13,14]. Advances in Wirtinger calculus and complex-valued backpropagation [15,16] have enabled stable training of such networks, leading to successful applications in signal processing, time-series analysis, image classification, and radar imaging [17,18]. Nevertheless, CVNNs often introduce additional training complexity and remain challenging to interpret, particularly when combined with deep, overparameterized architectures.

A recurring theme across these research directions is the need for neural models that balance interpretability, expressive power, and computational efficiency. Recent work has highlighted the role of activation functions as a key design axis for achieving this balance, motivating the development of parameterized and structure-aware nonlinearities [19]. In this context, aggregation-based operators offer a promising alternative to conventional pointwise activations by enabling higher-order feature interactions within individual units.

The present work contributes to this emerging direction by introducing LAUs, which combine adaptive aggregation, intrinsic interpretability, and compatibility with both real-valued and complex-valued representations. By grounding the activation function in the mathematically well-established Lehmer transform [1], the proposed approach provides a principled mechanism for feature weighting and nonlinear transformation. In contrast to prior interpretable or complex-valued models that rely on depth or post-hoc analysis, our approach emphasizes compact architectures in which interpretability is built directly into the computational primitive. This positions LAUs as a complementary contribution to existing interpretable and complex-valued neural network paradigms.

## 3. Mathematical Framework

### 3.1. Lehmer Transform

The Lehmer transform has been introduced as a parameterized family of means, capturing a spectrum of aggregation behaviors [1]. Let $x={\left[x_{1},\ldots ,x_{n}\right]}^{T}\in R_{>0}^{n}$ denote *n* strictly positive input features. For a parameter s∈R, the Lehmer transform is defined as

(1) $$L\left(s\right)=\frac{\sum_{i=1}^{n}x_{i}^{s}}{\sum_{i=1}^{n}x_{i}^{s-1}}.$$

The order parameter *s*, referred to as the suddency moment, governs the emphasis placed on small or large elements of x. When s=0, the transform coincides with the harmonic mean, prioritizing smaller values. For s=1, it reduces to the arithmetic mean, and for s=2 it yields the contra-harmonic mean, which accentuates larger elements. More generally,

(2) $$\lim_{s\to -\infty }L\left(s\right)=\min_{i}\left\{x_{i}\right\},\;\lim_{s\to +\infty }L\left(s\right)=\max_{i}\left\{x_{i}\right\},$$

so that L(s) provides a continuous interpolation between min-like and max-like aggregation.

A useful representation of the Lehmer transform is obtained by introducing the normalized weights

(3) $$p_{i}\left(s\right)=\frac{x_{i}^{s-1}}{\sum_{j=1}^{n}x_{j}^{s-1}},\;i=1,\ldots ,n,$$

which satisfy $p_{i}\left(s\right)>0$ and $\sum_{i=1}^{n}p_{i}\left(s\right)=1$. With this notation,

(4) $$L\left(s\right)=\sum_{i=1}^{n}p_{i}\left(s\right)\;x_{i}.$$

This representation admits a natural interpretation in terms of attention-like aggregation. The coefficients $p_{i}\left(s\right)$ act as data-dependent importance weights that determine the contribution of each coordinate $x_{i}$ to the aggregate L(s). Unlike static weighting schemes, these coefficients are induced directly by the input through a power-law transformation controlled by the suddency moment *s*. As *s* increases, the distribution $\left\{p_{i}\left(s\right)\right\}$ becomes increasingly concentrated on larger values of $x_{i}$, effectively focusing the aggregation on dominant components. Conversely, for smaller or negative values of *s*, the weights emphasize smaller entries, shifting attention toward lower-tail behavior. In this sense, the Lehmer transform implements a single-parameter, input-adaptive attention mechanism in which feature relevance emerges intrinsically from the data rather than being imposed externally. This intrinsic attention-like behavior provides a principled explanation for the interpretability of Lehmer-based aggregation and motivates its use as a fundamental building block in neural architectures.

A fundamental property of the Lehmer transform is its monotonicity with respect to the suddency moment *s*. For strictly positive inputs, differentiation of (1) yields

(5) $$\frac{\partial }{\partial s}L\left(s\right)=\frac{\sum_{i=1}^{n}\sum_{j=i+1}^{n}\left(x_{i}-x_{j}\right){\left(x_{i}x_{j}\right)}^{s-1}\ln\;\left(\frac{x_{i}}{x_{j}}\right)}{{\left(\sum_{i=1}^{n}x_{i}^{s-1}\right)}^{2}}.$$

**Figure 1:**
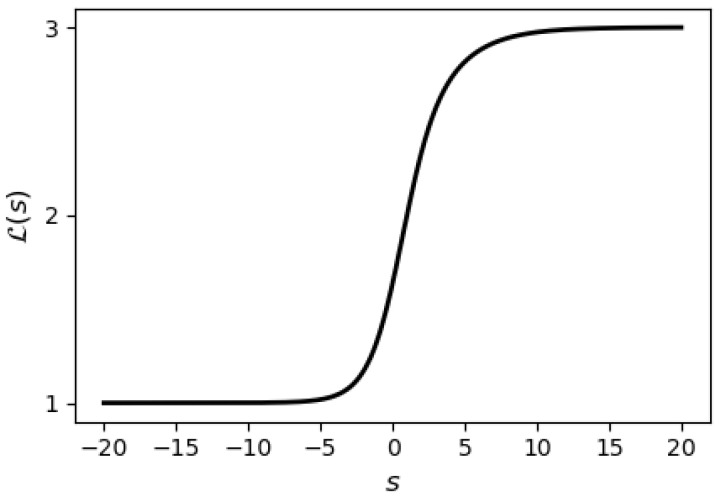
Lehmer transform L(s) as a function of *s* for $x={\left[1,2,3\right]}^{⊤}$.

For $x_{i},x_{j}>0$, the inequality

$$\left(x_{i}-x_{j}\right)\ln\;\left(\frac{x_{i}}{x_{j}}\right)\geq 0$$

holds, with equality if and only if $x_{i}=x_{j}$. Since ${\left(x_{i}x_{j}\right)}^{s-1}>0$, every unequal pair contributes positively to the numerator of (5). Consequently, L(s) is strictly increasing in *s* whenever not all components of x are equal. This monotonicity implies the ordering

(6) $$\min_{i}\left\{x_{i}\right\}\leq L\left(s\right)\leq L\left(t\right)\leq \max_{i}\left\{x_{i}\right\},$$

whenever s≤t. Figure 1 illustrates the strict monotonicity of the Lehmer transform L(s) as a function of the suddency moment *s* for the input vector $x={\left[1,2,3\right]}^{⊤}$.

In addition to monotonicity with respect to the suddency moment, the Lehmer transform exhibits a well-characterized behavior under majorization. Specifically, it is Schur-convex for s≥1 and Schur-concave for s≤1 (see, e.g., [20,21]). This distinction formalizes how the transform responds to the dispersion structure of the input vector. When s≥1, Schur-convexity implies that for any two vectors x and y with x majorizing y (i.e., x is more heterogeneous),

L(s;x)≥L(s;y),

so that increased heterogeneity leads to larger aggregated responses. Conversely, when s≤1, Schur-concavity implies the reverse ordering, meaning that greater dispersion suppresses the aggregated output. This dual behavior reveals that the suddency moment *s* governs not only the emphasis on large versus small components, but also whether the aggregation mechanism amplifies or attenuates feature heterogeneity.

From a modeling perspective, this property endows the Lehmer transform with a structurally adaptive inductive bias. For s≥1, the transform favors representations in which dominant features are amplified, making it suitable for settings where salient components should drive the response. For s≤1, the transform promotes robustness by emphasizing weaker components and discouraging excessive dominance. Unlike uniform averaging schemes, which are insensitive to the dispersion structure of the input, the Lehmer transform learns, through the suddency moment, whether heterogeneity should be preserved or suppressed. This yields an aggregation mechanism that is both adaptive and structurally interpretable, allowing neural representations to reflect the intrinsic organization of the data.

### 3.2. Weighted Lehmer Transform

The weighted Lehmer transform extends the Lehmer transform by incorporating positive weights $w={\left[w_{1},\ldots ,w_{n}\right]}^{T}\in R_{>0}^{n}$ as follows:

(7) $$L\left(s\right)=\frac{\sum_{i=1}^{n}w_{i}x_{i}^{s}}{\sum_{i=1}^{n}w_{i}x_{i}^{s-1}}.$$

The weights $w_{i}$ act as trainable parameters that modulate the relative importance of the input coordinates. Defining

(8) $$\pi_{i}\left(s\right)=\frac{w_{i}x_{i}^{s-1}}{\sum_{j=1}^{n}w_{j}x_{j}^{s-1}},$$

it follows that $\pi_{i}>0$, $\sum_{i}\pi_{i}=1$, and

(9) $$L\left(s\right)=\sum_{i=1}^{n}\pi_{i}\left(s\right)\;x_{i}.$$

Hence, the weighted Lehmer transform remains an explicitly interpretable aggregation, with $\pi_{i}$ providing sample-wise attribution scores.

The weighted transform is homogeneous of degree one in the inputs; i.e.,

L(s;λx,w)=λL(s;x,w),λ>0,

and invariant under proportional scaling of the weights; i.e.,

L(s;x,αw)=L(s;x,w),α>0.

It is also permutation invariant with respect to paired input–weight indices $\left(x_{i},w_{i}\right)$. These invariance properties endow the weighted Lehmer transform with a structurally consistent aggregation behavior. Homogeneity of degree one ensures that uniform rescaling of the inputs results in a proportional rescaling of the output, so the transform captures relative structure rather than absolute magnitude distortions. Invariance under proportional scaling of the weights implies that only their relative proportions affect the aggregation, eliminating scale-based identifiability ambiguities and allowing the weights to be interpreted as intrinsic importance coefficients. Permutation invariance with respect to paired input–weight indices further guarantees that the transform depends solely on the multiset of feature–weight pairs, not on their ordering. Together, these properties ensure that the weighted Lehmer transform provides a scale-aware and order-independent aggregation mechanism, making it well suited for robust and compact neural representations.

For fixed w>0, the limiting behavior remains unchanged as follows:

$$\lim_{s\to -\infty }L\left(s\right)=\min_{i}\left\{x_{i}\right\},\;\lim_{s\to +\infty }L\left(s\right)=\max_{i}\left\{x_{i}\right\}.$$

Differentiation with respect to *s* yields

(10) $$\frac{\partial }{\partial s}L\left(s\right)=L\left(s\right)\left(\frac{\sum_{i=1}^{n}w_{i}x_{i}^{s}\ln x_{i}}{\sum_{i=1}^{n}w_{i}x_{i}^{s}}-\frac{\sum_{i=1}^{n}w_{i}x_{i}^{s-1}\ln x_{i}}{\sum_{i=1}^{n}w_{i}x_{i}^{s-1}}\right),$$

which may be interpreted as a difference of logarithmic moments under two normalized measures.

Finally, the derivative with respect to a weight $w_{k}$ is

(11) $$\frac{\partial }{\partial w_{k}}L\left(s\right)=\frac{x_{k}^{s-1}}{\sum_{i=1}^{n}w_{i}x_{i}^{s-1}}\left(x_{k}-L\left(s\right)\right).$$

Thus, gradient-based learning increases $w_{k}$ when $x_{k}$ exceeds the current aggregate and decreases it otherwise, providing a transparent adjustment mechanism.

The weighted Lehmer transform therefore preserves the smoothness, monotonicity, and dispersion sensitivity of the unweighted case, while introducing adaptive feature modulation suitable for integration into neural architectures.

### 3.3. Complex-Valued Lehmer Transform

The Lehmer transform admits a natural extension to the complex domain, which substantially enriches its expressive capacity while preserving its fundamental aggregation structure. In this setting, the suddency moment is generalized from a real scalar to a complex parameter s=a+bi, where a,b∈R. The real part a=Re(s) retains its role as a scale-controlling parameter, whereas the imaginary part b=Im(s) introduces oscillatory behavior through complex exponentiation.

For strictly positive inputs $x\in R_{>0}^{n}$ and positive weights $w\in R_{>0}^{n}$, the complex-valued weighted Lehmer transform L:C→C is defined by

(12) $$L\left(s\right)=\frac{\sum_{i=1}^{n}w_{i}x_{i}^{a}e^{\;bi\ln(x_{i})}}{\sum_{i=1}^{n}w_{i}x_{i}^{a-1}e^{\;bi\ln(x_{i})}}.$$

This formulation preserves the ratio-of-moments structure of the real-valued Lehmer transform while allowing complex modulation through the logarithmic phase factors $e^{bi\ln(x_{i})}$. The positivity of $x_{i}$ ensures that $\ln(x_{i})$ is real-valued, avoiding branch ambiguities and guaranteeing a well-defined extension.

Using Euler’s identity, the transform may be equivalently expressed as

(13) $$L\left(s\right)=\frac{\sum_{i=1}^{n}w_{i}x_{i}^{a}\left(\cos\;\left(b\ln(x_{i})\right)+i\sin\;\left(b\ln(x_{i})\right)\right)}{\sum_{i=1}^{n}w_{i}x_{i}^{a-1}\left(\cos\;\left(b\ln(x_{i})\right)+i\sin\;\left(b\ln(x_{i})\right)\right)}.$$

This representation makes explicit the real and imaginary contributions induced by the imaginary component of *s*. The terms $\cos(b\ln\left(x_{i}\right))$ and $\sin(b\ln\left(x_{i}\right))$ act as phase factors that modulate the contribution of each input in logarithmic space.

Specifically, the imaginary component *b* introduces interference effects through the logarithmic phases $b\ln(x_{i})$. Each term $x_{i}^{s}=x_{i}^{a}e^{ib\ln(x_{i})}$ contributes a complex phasor whose phase is proportional to $\ln(x_{i})$, so that the numerator and denominator of (12) form superpositions of such phasors. When phases are approximately aligned across inputs, the corresponding contributions reinforce one another, yielding constructive interference and an amplified aggregate response. Conversely, phase misalignment produces destructive interference and partial cancellation. In particular, cancellation between two components becomes pronounced when

$$b\left(\ln\left(x_{i}\right)-\ln\left(x_{j}\right)\right)\equiv \pi \;\left(\mathrm{mod}\;2\pi \right),$$

demonstrating that the transform is explicitly sensitive to logarithmic ratios rather than solely to absolute magnitudes. This phase-sensitive mechanism enables the encoding of fine-grained relational structure among inputs that is inaccessible to purely real-valued aggregation.

The explicit dependence on $\ln(x_{i})$ situates the transform naturally within a logarithmic geometry. Both the magnitude modulation via $x_{i}^{a}$ and the phase modulation via $e^{ib\ln(x_{i})}$ operate intrinsically in log-space, making the transform well adapted to data exhibiting multiplicative structure, scale variability, or power-law behavior. When the dispersion of $\ln(x_{i})$ is small, phase differences remain limited and the transform behaves similarly to its real-valued counterpart, with aggregation dominated by magnitude effects. As the spread of $\ln(x_{i})$ increases, phase diversity becomes more pronounced and the aggregation increasingly reflects higher-order relational structure through interference patterns.

The joint action of the real and imaginary components of *s* thus yields a dual sensitivity: the real part *a* governs scale-based aggregation, controlling the relative emphasis on larger versus smaller values, while the imaginary part *b* encodes relative logarithmic differences through phase modulation. This combination produces a flexible and interpretable aggregation mechanism capable of simultaneously capturing global magnitude trends and localized relational organization. Such properties make the complex-valued Lehmer transform particularly well suited for structured or heterogeneous data, where both scale and relative organization play a fundamental role.

From an analytical standpoint, the complex-valued Lehmer transform may be written as L(s)=N(s)/N(s−1), where

$$N\left(s\right)=\sum_{i=1}^{n}w_{i}e^{s\ln(x_{i})}.$$

Since N(s) is an entire function of *s*, the transform is meromorphic on C, with singularities arising only at isolated zeros of the denominator D(s)=N(s−1). Writing s=a+ib, $t_{i}=\ln\left(x_{i}\right)$, and $c_{i}\left(a\right)=w_{i}x_{i}^{a-1}>0$, the denominator admits the phasor representation

$$D\left(a+ib\right)=\sum_{i=1}^{n}c_{i}\left(a\right)e^{ibt_{i}}.$$

Hence, D(s)=0 if and only if the complex vectors $c_{i}\left(a\right)e^{ibt_{i}}$ cancel exactly, equivalently when

$$\sum_{i=1}^{n}c_{i}\left(a\right)\cos\left(bt_{i}\right)=0,\;\sum_{i=1}^{n}c_{i}\left(a\right)\sin\left(bt_{i}\right)=0$$

are simultaneously satisfied. Such configurations correspond to precise phase cancellation across inputs and are structurally non-generic. Since D(s) is a nonconstant entire function whenever the inputs are not all identical, its zeros are isolated in C, implying that the singular set of L(s) forms a discrete subset of the (a,b)-plane and therefore has Lebesgue measure zero in $R^{2}$, provided the inputs are not all equal. Exact poles are thus non-generic under continuous optimization.

Nevertheless, near-cancellation of the denominator may occur when |D(s)| becomes small, leading to large magnitudes of the transform and amplified gradients due to the quotient structure. For any parameter $\theta \in \{a,b,w_{1},\ldots ,w_{n}\}$, the derivative satisfies

$$\frac{\partial L}{\partial \theta }=\frac{\left(\partial_{\theta }N\right)D-N\left(\partial_{\theta }D\right)}{D^{2}},$$

so gradient magnitudes can grow on the order of ${\left|D\left(s\right)\right|}^{-2}$ near cancellation. This regime corresponds to highly phase-selective behavior, where small perturbations in (a,b) or in the logarithmic geometry of the inputs induce large changes in the aggregate. In practice, numerical stability can be ensured through standard techniques such as safe division (e.g., D↦D+ε with small ε>0), mild constraints on the magnitude of *b*, or normalization strategies that prevent extreme dynamic ranges in the coefficients $c_{i}\left(a\right)$.

Lastly, differentiability with respect to the real and imaginary components of *s* follows directly. Using Wirtinger calculus, the derivative with respect to the complex parameter may be written as

(14) $$\frac{\partial L}{\partial s}=\frac{\partial L}{\partial a}+i\;\frac{\partial L}{\partial b},$$

where ∂L/∂a governs sensitivity to scale-based variations and ∂L/∂b controls sensitivity to oscillatory, phase-like effects. This decomposition provides a clear separation between magnitude-driven and phase-driven learning dynamics.

## 4. Lehmer Activation Units

The aggregation properties of the Lehmer transform developed in Section 3 naturally motivate its use as a nonlinear computational primitive in neural networks. Rather than acting elementwise, Lehmer-based activations operate on collections of features and produce adaptive aggregates whose behavior is governed by learnable parameters. We refer to these operators as Lehmer Activation Units (LAUs). An LAU simultaneously determines (i) how strongly each input feature contributes to the output and (ii) how those contributions are aggregated, thereby unifying feature weighting and nonlinearity in a single differentiable mechanism.

A defining characteristic of LAUs is that their nonlinearity is not fixed a priori, but is instead learned from data through the suddency parameter and the feature weights. As a result, the expressive power of an LAU exceeds that of conventional pointwise activations, while retaining interpretability through explicit aggregation structure.

### 4.1. Real-Valued Lehmer Activation Unit

The real-valued LAU is obtained by embedding the weighted Lehmer transform into a neural activation. Let $x\in R_{>0}^{n}$ denote the input to the unit, $w\in R_{>0}^{n}$ a vector of trainable weights, and s∈R a trainable suddency moment. The activation is then defined as follows:

(15) $$\mathrm{RLAU}\left(x;w,s\right)=\frac{\sum_{i=1}^{n}w_{i}x_{i}^{\;s}}{\sum_{i=1}^{n}w_{i}x_{i}^{\;s-1}}.$$

When *s* varies, the real-valued LAU (15) continuously interpolates between different aggregation regimes (harmonic-like, arithmetic-like, contra-harmonic-like), and in the limiting sense approaches min-like and max-like behavior. Consequently, the nonlinearity of a single LAU is not predetermined but learned from data by jointly optimizing w and *s*, allowing each unit to adapt its response to the statistics of the task.

To ensure well-definedness in neural settings, LAUs must accommodate general real pre-activations while preserving the strict positivity required by the Lehmer transform. This is achieved via a smooth positivity-preserving embedding applied upstream of (15). A standard choice consistent with our implementations is a sign-preserving positive embedding

$$x\in R^{n}\;\mapsto \;u\in R_{>0}^{2n},\;u=\left(\phi \left(x_{1}\right),\ldots ,\phi \left(x_{n}\right),\phi \left(-x_{1}\right),\ldots ,\phi \left(-x_{n}\right)\right),\;\phi \left(t\right)=\mathrm{softplus}\left(\kappa t\right),$$

which retains sign information while ensuring differentiability and avoiding logarithmic singularities. Here, κ is a trainable parameter.

The softplus-based embedding is a smooth, strictly monotone transformation applied component-wise. Consequently, it preserves ordering relations within each coordinate and does not mix features across dimensions. From a geometric perspective, the embedding can therefore be interpreted as a reparameterization of the input space rather than as a feature-mixing transformation.

Importantly, the role of this embedding is primarily technical. It guarantees that the Lehmer operator is well-defined over the entire training domain and remains compatible with gradient-based optimization, while avoiding degenerate behavior associated with nonpositive inputs. Since the transformation is monotone and applied independently to each coordinate, it does not by itself introduce additional expressive power, but instead provides a stable and analytically consistent interface between unconstrained neural pre-activations and the positivity requirements of the Lehmer transform.

In addition, weights are enforced to remain positive using smooth parameterizations such as

$$w_{i}=\ln\left(1+e^{v_{i}}\right),$$

where $v_{i}$ are unconstrained training parameters. To ensure numerical stability, LAUs further incorporate input standardization to a predefined range, such as $\left(e^{-1},e\right)$, ensuring well-conditioned computations and robust training performance.

To illustrate the interpretability of LAUs, we provide the following example, which demonstrates how analyzing the learned network parameters can reveal the underlying mechanisms guiding the decision-making process.

**Example** **1.** *We consider a neural network with a single hidden layer consisting of three real-valued LAUs, trained on the Iris dataset. After training, the learned weight matrix of the Lehmer layer is*

$$W=\left[\begin{array}{ccc} 0.64 & 0.07 & 0.01\\ 0.32 & 3.04 & 0.03\\ 1.47 & 0.03 & 2.84\\ 0.60 & 0.01 & 0.49 \end{array}\right],$$

*where rows correspond to the input features (sepal length, sepal width, petal length, and petal width) and columns correspond to individual LAUs. The learned suddency moments for the three units are*

$$s={\left[-1.81,\;0.32,\;2.42\right]}^{⊤}.$$

*The first unit, with s=−1.81, operates in a regime that emphasizes smaller weighted inputs. Its weight profile assigns dominant importance to petal length and petal width. This configuration yields sensitivity to small petal measurements, a defining characteristic of the Setosa class, indicating that the unit functions as a detector of compact petal structure.*

*The second unit, with s=0.32, lies between harmonic and arithmetic aggregation. Its dominant weight is associated with sepal width, suggesting that this unit captures variations in sepal morphology that contribute to discrimination between Setosa and the remaining species. The intermediate value of s reflects a balanced aggregation that neither suppresses nor overly amplifies large inputs.*

*The third unit, with s=2.42, operates in a regime that accentuates larger weighted inputs. It places strong emphasis on petal length and a secondary emphasis on petal width, aligning with the larger petal dimensions characteristic of the Virginica class. This unit therefore acts as a detector of expansive petal structure.*

*Overall, the learned parameters demonstrate that each LAU simultaneously encodes what features are important through its weights and how those features are aggregated through its suddency moment. The resulting representations are directly interpretable and closely aligned with known morphological distinctions in the Iris dataset.*

### 4.2. Expressivity and Universal Approximation

We now formalize the expressive power of real-valued Lehmer Activation Units by proving a universal approximation theorem. The key technical ingredient is that a single two-input RLAU can uniformly approximate the max operator on compact positive domains. This property allows RLAUs to emulate the ReLU nonlinearity with arbitrary precision, and thereby inherit the classical universality of shallow ReLU networks.

We begin with a quantitative approximation bound for the Lehmer transform.

**Lemma** **1** (Max Approximation by Lehmer Transform)**.** *Fix $0<m_{0}\leq m_{1}<\infty$ and let $u,v\in [m_{0},m_{1}]$. For s>1, define*

(16) $$L\left(s;u,v\right):=\frac{u^{s}+v^{s}}{u^{s-1}+v^{s-1}}.$$

*Then for all $\left(u,v\right)\in {\left[m_{0},m_{1}\right]}^{2}$,*

(17) $$0\leq \max\left\{u,v\right\}-L\left(s;u,v\right)\leq \frac{m_{1}}{e\;s}.$$

*In particular, L(s;u,v)→max{u,v} uniformly on ${\left[m_{0},m_{1}\right]}^{2}$ as s→∞.*

**Proof.**  Let M=max{u,v} and m=min{u,v}, and define r:=m/M∈(0,1]. Factoring $M^{s-1}$ from the numerator and denominator in (16) yields

(18) $$L\left(s;u,v\right)=M\frac{1+r^{s}}{1+r^{s-1}}.$$

Since r∈(0,1] and s>1, we have $r^{s}\leq r^{s-1}$, hence

(19) $$\frac{1+r^{s}}{1+r^{s-1}}\leq 1\;⟹\;L\left(s;u,v\right)\leq M,$$

which implies 0≤M−L(s;u,v).

Moreover, from (18),

(20) $$M-L\left(s;u,v\right)=M\left(1-\frac{1+r^{s}}{1+r^{s-1}}\right)=M\frac{r^{s-1}-r^{s}}{1+r^{s-1}}=M\frac{r^{s-1}\left(1-r\right)}{1+r^{s-1}}\leq Mr^{s-1}\left(1-r\right).$$

Since $M\leq m_{1}$, it suffices to control $\sup_{r\in (0,1]}r^{s-1}\left(1-r\right)$.

Define $f\left(r\right)=r^{s-1}\left(1-r\right)$ on (0,1]. Then

(21) $$f^{\prime }\left(r\right)=r^{s-2}\left((s-1)-sr\right),$$

so the unique maximizer is $r^{*}=\left(s-1\right)/s$. Evaluating at $r^{*}$ gives

(22) $$\underset{r\in (0,1]}{\sup}f\left(r\right)=f\left(r^{*}\right)={\left(\frac{s-1}{s}\right)}^{s-1}\frac{1}{s}={\left(1-\frac{1}{s}\right)}^{s-1}\frac{1}{s}\leq \frac{1}{e\;s},$$

where we used ${\left(1-\frac{1}{s}\right)}^{s-1}\leq e^{-1}$ for all s>1. Combining (20) and (22) yields the desired inequality (17). □

Lemma 1 shows that a two-input RLAU approximates the max operator uniformly on compact positive domains, with error decaying at rate O(1/s). This observation allows us to approximate the ReLU nonlinearity on compact domains using shifted Lehmer transforms.

**Lemma** **2** (Uniform ReLU Approximation)**.** *Let $K\subset R^{d}$ be compact, $\ell \left(x\right)=v^{⊤}x+t$ affine, and σ(z)=max{0,z}. Fix $m_{0}>0$ and choose C>0 such that*

(23) $$C\geq m_{0},\;\ell \left(x\right)+C\geq m_{0}\;\forall x\in K.$$

*Define*

(24) $$\sigma_{s,C}\left(x\right):=L\left(s;\;C,\;\ell (x)+C\right)-C.$$

*Let*

(25) $$m_{1}:=\left(\underset{x\in K}{\sup}\left(\ell \left(x\right)+C\right)\right)\vee C<\infty .$$

*Then for all s>1,*

(26) $$\underset{x\in K}{\sup}|\sigma \left(\ell \left(x\right)\right)-\sigma_{s,C}\left(x\right)|\leq \frac{m_{1}}{e\;s}.$$

**Proof.**  Since *ℓ* is continuous and *K* is compact, *ℓ* is bounded below on *K*, so there exists C>0 satisfying (23). For any x∈K,

(27) σ(ℓ(x))=max{0,ℓ(x)}=max{C,ℓ(x)+C}−C.

By construction, for all x∈K we have $\left(C,\ell \left(x\right)+C\right)\in {\left[m_{0},m_{1}\right]}^{2}$. Applying Lemma 1 to (u,v)=(C,ℓ(x)+C) yields

(28) $$0\leq \max\left\{C,\ell \left(x\right)+C\right\}-L\left(s;C,\ell \left(x\right)+C\right)\leq \frac{m_{1}}{e\;s}.$$

Subtracting *C* from both sides of (28) and using (27) gives

(29) $$0\leq \sigma \left(\ell \left(x\right)\right)-\sigma_{s,C}\left(x\right)\leq \frac{m_{1}}{e\;s}\;\forall x\in K.$$

Taking the supremum over x∈K in (29) yields (26). □

Lemmas 1 and 2 together establish that individual RLAUs can uniformly approximate ReLU activations on compact sets. We now lift this approximation from individual neurons to entire shallow networks, yielding a universal approximation theorem.

**Theorem** **1** (Universal Approximation Theorem)**.** *Let $K\subset R^{d}$ be compact and let f∈C(K). Assume the classical universal approximation theorem for one-hidden-layer ReLU networks: for every ε>0 there exist M∈N, affine maps $\ell_{j}\left(x\right)=v_{j}^{⊤}x+t_{j}$, coefficients $a_{j}\in R$, and b∈R such that*

(30) $$f_{\mathrm{ReLU}}\left(x\right)=\sum_{j=1}^{M}a_{j}\;\sigma \left(\ell_{j}\left(x\right)\right)+b,\;\underset{x\in K}{\sup}\left|f\left(x\right)-f_{\mathrm{ReLU}}\left(x\right)\right|<\varepsilon /2.$$

*Then for every ε>0 there exist shifts $C_{j}>0$ and parameters $s_{j}>1$ such that the one-hidden-layer RLAU network*

(31) $$F\left(x\right)=\sum_{j=1}^{M}a_{j}\left(L\left(s_{j};\;C_{j},\;\ell_{j}\left(x\right)+C_{j}\right)-C_{j}\right)+b$$

*satisfies*

(32) $$\underset{x\in K}{\sup}\left|f\left(x\right)-F\left(x\right)\right|<\varepsilon .$$

**Proof.**  Fix ε>0 and choose $f_{\mathrm{ReLU}}$ satisfying (30). Define

(33) $$A:=\sum_{j=1}^{M}\left|a_{j}\right|.$$

Fix any $m_{0}>0$. For each *j*, continuity of $\ell_{j}$ and compactness of *K* imply $\inf_{x\in K}\ell_{j}\left(x\right)>-\infty$, hence there exists $C_{j}>0$ such that

(34) $$C_{j}\geq m_{0},\;\ell_{j}\left(x\right)+C_{j}\geq m_{0}\;\forall x\in K.$$

Define, for each *j*,

(35) $$\sigma_{j}\left(x\right):=L\left(s_{j};\;C_{j},\;\ell_{j}\left(x\right)+C_{j}\right)-C_{j},$$

and let

(36) $$m_{1,j}:=\left(\underset{x\in K}{\sup}\left(\ell_{j}\left(x\right)+C_{j}\right)\right)\vee C_{j}<\infty .$$

By Lemma 2,

(37) $$\underset{x\in K}{\sup}|\sigma \left(\ell_{j}\left(x\right)\right)-\sigma_{j}\left(x\right)|\leq \frac{m_{1,j}}{e\;s_{j}}.$$

Choose $s_{j}>1$ sufficiently large such that

(38) $$\frac{m_{1,j}}{e\;s_{j}}<\frac{\varepsilon }{2M\;(A\vee 1)}.$$

Define *F* by (31). Then for all x∈K,

(39) $$|f_{\mathrm{ReLU}}\left(x\right)-F\left(x\right)|\leq \sum_{j=1}^{M}|a_{j}|\;|\sigma \left(\ell_{j}\left(x\right)\right)-\sigma_{j}\left(x\right)|\leq \sum_{j=1}^{M}|a_{j}|\underset{x\in K}{\sup}\left|\sigma \left(\ell_{j}\left(x\right)\right)-\sigma_{j}\left(x\right)\right|.$$

Combining (39) with (38) gives

(40) $$|f_{\mathrm{ReLU}}\left(x\right)-F\left(x\right)|<\frac{\varepsilon }{2}\cdot \frac{A}{A\vee 1}\leq \frac{\varepsilon }{2}.$$

Finally,

(41) $$\underset{x\in K}{\sup}\left|f\left(x\right)-F\left(x\right)\right|\leq \underset{x\in K}{\sup}|f\left(x\right)-f_{\mathrm{ReLU}}\left(x\right)|+\underset{x\in K}{\sup}\left|f_{\mathrm{ReLU}}\left(x\right)-F\left(x\right)\right|<\varepsilon ,$$

which proves (32). □

Theorem 1 establishes that one-hidden-layer RLAU networks are universal approximators on compact domains. Notably, this universality does not rely on piecewise linearity, but instead arises from the adaptive aggregation structure of the Lehmer transform. As the suddency parameter *s* increases, each RLAU interpolates smoothly toward a max-like nonlinearity, while for moderate *s* it retains a smooth, mean-like character. Thus, RLAUs provide a principled continuum between linear pooling and highly nonlinear maxout-style responses, allowing expressive power to be tuned continuously by the model rather than imposed architecturally.

### 4.3. Complex-Valued Lehmer Activation Unit

The complex-valued Lehmer activation unit (CLAU) extends the real-valued LAU by allowing the suddency moment to take complex values s=a+bi∈C, where a,b∈R are trainable parameters. For positive inputs $x\in R_{>0}^{n}$ and positive weights $w\in R_{>0}^{n}$, the complex Lehmer activation is defined by

(42) $$\mathrm{CLAU}\left(x;w,a,b\right)=\frac{\sum_{i=1}^{n}w_{i}x_{i}^{a}e^{\;bi\ln(x_{i})}}{\sum_{i=1}^{n}w_{i}x_{i}^{a-1}e^{\;bi\ln(x_{i})}}\in C.$$

Equivalently, using Euler’s identity,

$$x_{i}^{a}e^{\;bi\ln(x_{i})}=x_{i}^{a}\left(\cos\left(b\ln x_{i}\right)+i\sin\left(b\ln x_{i}\right)\right),$$

which shows explicitly how the imaginary component induces oscillatory modulation in logarithmic space.

The real part a=Re(s) retains the magnitude-selective role of the real-valued LAU, governing the transition between regimes that emphasize small versus large values. The imaginary part b=Im(s) introduces phase factors $\cos(b\ln x_{i})$ and $\sin(b\ln x_{i})$, producing constructive and destructive interference among terms in the numerator and denominator of (42). This mechanism enables the unit to encode relational structure among features that depends on their relative logarithmic magnitudes, thereby expanding the representational capacity beyond purely real aggregation.

To integrate CLAUs into standard deep networks that require real-valued activations for normalization layers and loss functions, the complex output *z* is mapped to a real scalar through a structured readout. In our scheme, we use the differentiable mapping

(43) ReCLAU(z)=αRe(z)+βIm(z)+γ|z|2+δ,

where α,β,γ,δ∈R are trainable parameters defined per unit. This readout preserves information from both real and imaginary components while ensuring compatibility with standard objectives such as cross-entropy and with real-valued architectural components. The resulting CLAU mapping is smooth in the parameters (a,b) and fully compatible with gradient-based optimization, where the decomposition into (a,b) provides a natural separation between magnitude-driven and phase-driven adaptation.

The specific form of the readout in (43) is not unique, but represents a simple and principled choice that balances expressivity, numerical stability, and interpretability. The combination of real, imaginary, and magnitude-dependent terms provides sufficient flexibility to exploit complex-valued aggregation without introducing additional architectural complexity.

### 4.4. Normalization, Pooling, and Attention in LAU Networks

LAU-based networks are compatible with standard architectural operators. Let $h\in R^{B\times C\times H\times W}$ denote a real-valued feature map produced by either RLAU units directly or CLAU units followed by the real-valued readout (43). Normalization layers stabilize training by controlling the scale and dispersion of activations while preserving the functional role of LAU aggregation. Batch normalization standardizes each channel using batch and spatial statistics,

$$\mu_{c}=\frac{1}{BHW}\sum_{b,i,j}h_{b,c,i,j},\;\sigma_{c}^{2}=\frac{1}{BHW}\sum_{b,i,j}{\left(h_{b,c,i,j}-\mu_{c}\right)}^{2},$$

followed by a learned affine map. Group normalization performs an analogous standardization over channel groups and is independent of batch size, which can be beneficial when LAU models are trained with small batches. For vector-valued representations (e.g., after global pooling or in dense LAU heads), layer normalization standardizes across feature coordinates within each sample. These operators do not alter the permutation-invariant aggregation semantics of LAUs; rather, they regulate the amplitude and conditioning of the aggregated responses.

Pooling operators provide spatial aggregation on top of the intrinsic feature aggregation performed within each LAU. Local average pooling aggregates within spatial neighborhoods $\Omega_{i,j}$,

$${\left(\mathrm{AvgPool}\;h\right)}_{b,c,i,j}=\frac{1}{|\Omega_{i,j}|}\sum_{\left(p,q\right)\in \Omega_{i,j}}h_{b,c,p,q},$$

while max pooling selects the dominant response in each neighborhood. Global average pooling (GAP) collapses spatial dimensions entirely,

$${\left(\mathrm{GAP}\;h\right)}_{b,c}=\frac{1}{HW}\sum_{i,j}h_{b,c,i,j},$$

yielding compact descriptors that are particularly natural when LAU layers are used as channel-wise feature extractors. In this sense, pooling may be viewed as a hierarchical aggregation mechanism layered over Lehmer-based aggregation: LAUs aggregate within receptive-field feature collections, whereas pooling aggregates across spatial positions.

Finally, attention mechanisms may be incorporated via channel-wise gating (e.g., squeeze-and-excitation). A squeeze operation computes channel statistics

$$s_{b,c}=\frac{1}{HW}\sum_{i,j}h_{b,c,i,j},$$

which are transformed by a low-dimensional nonlinear map and a sigmoid to produce gating coefficients $g_{b,c}\in \left(0,1\right)$. The feature map is then reweighted multiplicatively,

$${\tilde{h}}_{b,c,i,j}=g_{b,c}\;h_{b,c,i,j}.$$

This form of attention operates at the level of channels (feature maps) and is complementary to the intrinsic feature-level attention induced by the escort weights within each LAU. Together they yield a hierarchical attribution structure: LAUs determine how features within a unit are aggregated, while channel attention modulates the relative importance of entire aggregated feature maps.

Overall, LAUs provide a mathematically grounded alternative to pointwise nonlinearities by combining adaptive aggregation, interpretable weighting, and compatibility with standard deep learning operators. The real-valued LAU captures a continuum of magnitude-selective pooling behaviors, while the complex-valued LAU further introduces phase-like modulation and interference in logarithmic space, offering additional modeling flexibility for structured and heterogeneous data.

## 5. Computational Results

This section evaluates Lehmer Activation Units in both real-valued (RLAU) and complex-valued (CLAU) formulations on representative tabular and image-based classification benchmarks. The experimental design deliberately employs extremely constrained architectures in order to isolate the contribution of the activation mechanism itself, avoiding confounding effects from architectural depth, overparameterization, or auxiliary components.

### 5.1. Tabular Data

For tabular datasets, we employed a deliberately minimal architecture: a single dense hidden layer with exactly one activation unit, followed by a linear output layer. No normalization, regularization, architectural refinements, or auxiliary nonlinearities were used. RLAU and CLAU were evaluated alongside standard pointwise activations (ReLU, ELU, GELU, Swish, and Mish) under identical training conditions using stratified 10-fold cross-validation with 20 repeats.

Table 1 reports classification performance across all datasets. Despite the extremely restricted model capacity, both RLAU and CLAU achieve near-ceiling accuracy across all benchmarks. In particular, on Iris and Wine, a single Lehmer unit is sufficient to match or exceed the performance of substantially more expressive pointwise nonlinearities. On WBC, CLAU achieves the highest mean accuracy and the lowest variance, indicating that the complex-valued extension offers additional robustness in higher-dimensional and noisier settings. It is noteworthy that in the single-unit regime, pointwise activations (notably ReLU) frequently suffer from degenerate training dynamics (e.g., near-constant hidden activations), leading to high variance across resampling; this effect disappears with modest width.

These results provide direct empirical evidence that LAUs concentrate representational power within individual neurons: meaningful feature interactions are captured even in single-unit architectures, without reliance on depth or auxiliary architectural complexity.

Table 2 quantifies computational costs. Because all models share the same architecture, differences arise solely from the activation mechanism.

RLAU preserves the parameter count and introduces only mild arithmetic overhead. FLOPs increase by at most 25% on Iris and become negligible for higher-dimensional datasets (only 3% on WBC), indicating that the cost of Lehmer aggregation amortizes as input dimensionality grows. Runtime increases by a factor of 1.7–1.9, consistent with the added exponentiation and normalization operations.

CLAU introduces only a handful of additional scalar parameters, yet incurs higher arithmetic cost due to complex exponentiation and the nonlinear decoder. FLOPs increase by a factor of 1.2–2.6 and runtime by roughly 2.7–3.1×. Nevertheless, absolute runtimes remain sub-second across all datasets, while the gains in accuracy and stability (notably on WBC) justify the additional flexibility.

These results confirm that performance improvements stem from the functional form of the activation rather than increased parameterization.

**Figure 2:**
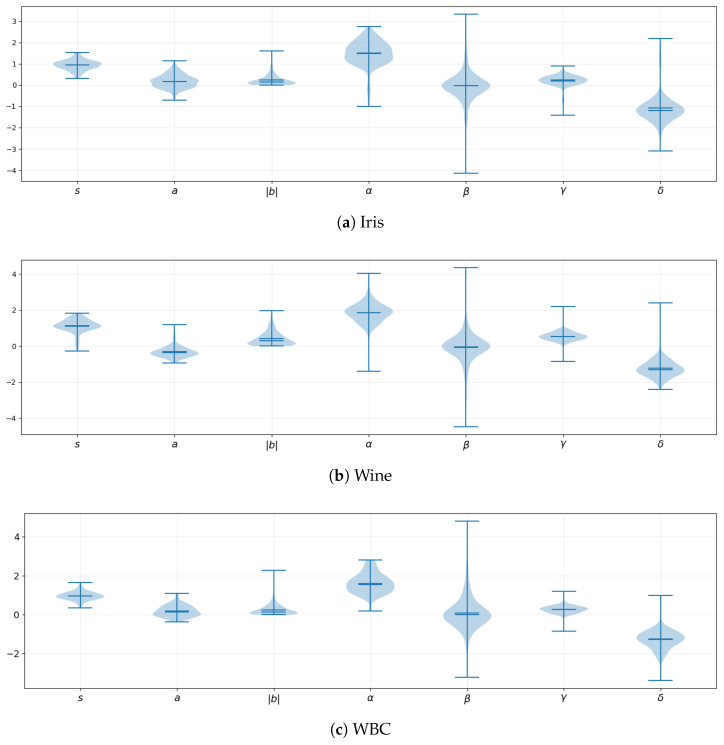
Violin plots of learned parameters across 200 splits.

Figure 2 reports the empirical distributions of the learned RLAU suddency moment *s* and CLAU parameters (a,|b|,α,β,γ,δ) across all 200 splits.

Across all datasets, the CLAU decoder consistently learns a strongly real-dominated readout: α concentrates around [1,2] while β remains centered near zero. Thus, the discriminative signal is carried primarily by ℜ(z), with ℑ(z) used selectively. The parameter γ is typically positive, so the term ${\gamma |z|}^{2}$ contributes a phase-invariant stabilizing component. The offset δ concentrates around negative values, functioning as a learned threshold.

On Iris (4 features), *s* concentrates well above 1, indicating a max-like aggregation regime consistent with the dominance of petal-related features. The parameter |b| is clearly non-negligible, indicating active exploitation of phase modulation and encoding of relative log-ratio structure among a small number of coordinates.

On Wine (13 features), *s* concentrates slightly above 1, corresponding to near-mean aggregation with mild selectivity. Phase effects are present but subdued, and β exhibits heavier tails, indicating occasional corrective use of the imaginary component.

On WBC (30 features), *s* concentrates tightly near 1, reflecting an averaging-like regime appropriate for high-dimensional noisy settings. Although |b| remains near zero, the heavy-tailed distribution of β indicates that CLAU selectively exploits phase-sensitive contributions when beneficial, explaining the observed variance reduction.

**Figure 3:**
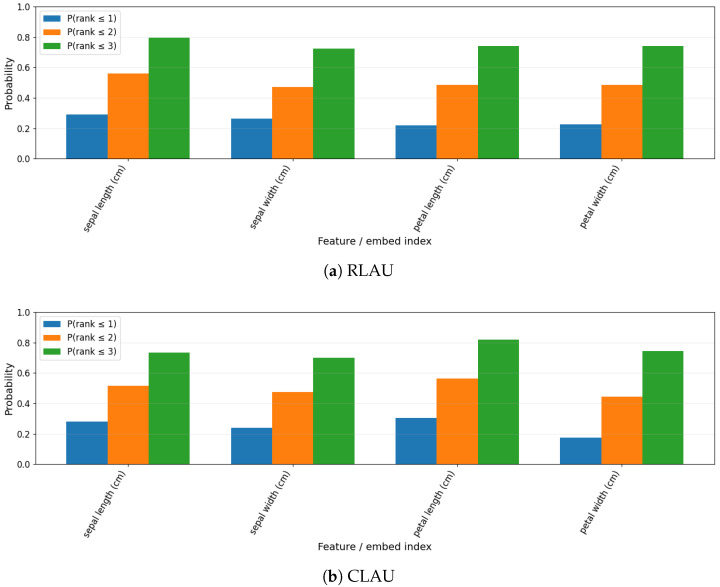
Feature-ranking stability on Iris.

Figure 3 reports feature-ranking stability on Iris. Both RLAU and CLAU consistently prioritize petal length and petal width across splits, closely matching the known discriminative structure of the dataset. CLAU exhibits slightly sharper separation between dominant and secondary features, consistent with its enhanced expressive flexibility. Importantly, attribution patterns are stable across resampling, confirming that interpretability is not an artifact of particular train–test splits.

**Figure 4:**
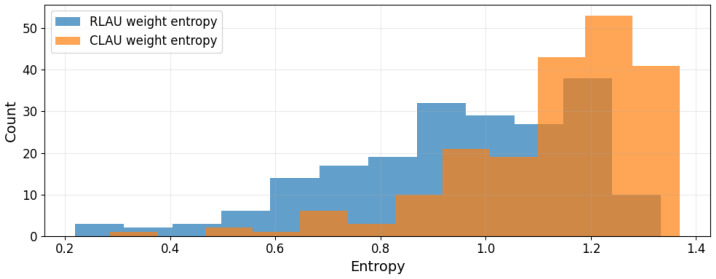
Entropy distributions of LAU weights across 200 splits on Iris.

Figure 4 reports the entropy distributions of normalized LAU weights on Iris. RLAU consistently exhibits lower entropy, indicating more concentrated attribution. CLAU yields a right-shifted entropy distribution, reflecting broader utilization of multiple features enabled by phase-sensitive interactions. This increase in entropy coincides with improved accuracy and stability, suggesting richer yet structured representation rather than diffuse noise.

**Figure 5:**
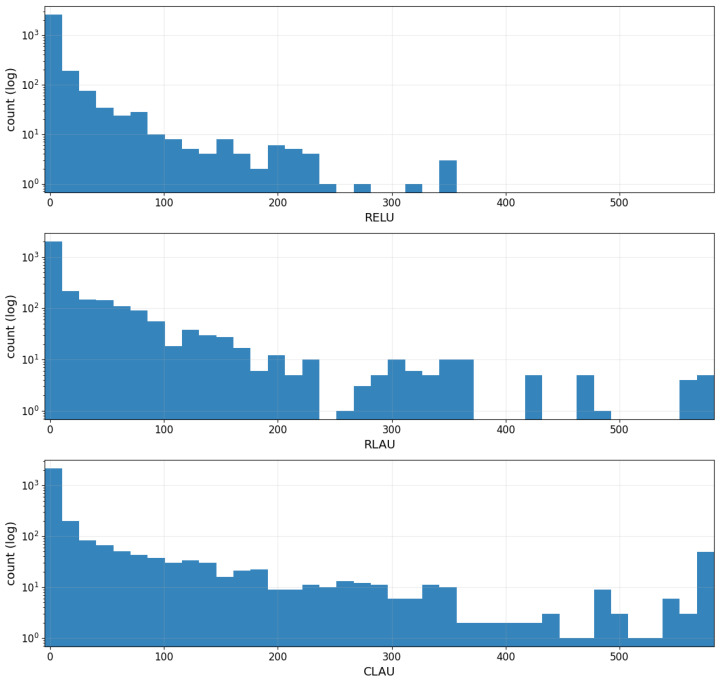
Activation/output histograms (log-count) on Iris.

Figure 5 reports histograms (log-count) of pre-classifier activations for ReLU, RLAU, and CLAU on Iris. ReLU produces highly concentrated outputs near zero, reflecting limited dynamic range and consistent with its weak performance in shallow settings. RLAU yields broader, heavier-tailed activations, indicating richer geometry induced by aggregation-based nonlinearities. CLAU further expands the activation range while remaining well-behaved, supporting the interpretation that complex-valued aggregation enhances representational richness rather than merely increasing variance.

### 5.2. Image-Based Data

For image benchmarks, Lehmer Activation Units are evaluated in a convolutional architecture where aggregation-based nonlinearities act only at the decision stage, while the convolutional backbone remains fully standard. This design isolates the effect of LAUs on final-stage aggregation and decision geometry, so observed differences can be attributed to the proposed activation mechanism rather than to changes in feature extraction.

All experiments use a WideResNet-28-10 backbone. Given an input image x, the backbone produces a feature vector $f\in R^{640}$ through residual convolutional blocks with BatchNorm and ReLU; the 640-dimensional representation is fixed across datasets because the same backbone configuration is used throughout. No LAUs are used inside convolutional layers. The decision module applies LayerNorm to f and then a compact Lehmer head consisting of a single RLAU or CLAU unit producing one scalar. This scalar is injected back into the representation via a residual projection and followed by a final linear classifier. Consequently, the classifier effectively relies on one aggregation-based neuron to summarize the backbone representation. The architecture is summarized in Table 3.

All models are trained using the same pipeline to ensure strict comparability between RLAU and CLAU. Optimization uses SGD with Nesterov momentum (0.9), initial learning rate 0.1 with cosine scheduling, and weight decay $5\times 10^{-4}$. We use batch size 128, automatic mixed precision, gradient clipping (norm 1.0), and exponential moving average (decay 0.999). Training is performed on a single NVIDIA A100 (40GB). The number of epochs is dataset-dependent: MNIST, EMNIST, KMNIST, FMNIST, and SVHN are trained for 10 epochs; CIFAR-10, CIFAR-100, and Tiny-ImageNet for 50 epochs. Moreover, the only dataset-specific component is the augmentation policy as reported in Table 4.

Table 5 reports classification accuracy. The results are notable because the decision stage uses only one aggregation-based neuron: the backbone must compress all class-discriminative evidence into $f\in R^{640}$, and the LAU head must then form a single informative scalar summary that, after residual injection, yields a linearly separable representation. Despite this extreme constraint, both variants achieve strong performance across diverse datasets. CLAU consistently improves over RLAU, suggesting that phase-sensitive modulation and the real-valued decoder provide additional modeling flexibility even when the backbone is fixed.

To quantify resource usage, we report parameter count, FLOPs, peak GPU memory, and time per epoch, obtained from profiler instrumentation on the GPU (Table 6). As expected, computational cost is dominated by the WideResNet backbone (about 5.25 G FLOPs for 32×32 inputs and 21.0 G for Tiny-ImageNet resolution). Since the Lehmer head is a single unit applied to a 640-dimensional vector, its incremental cost is negligible at this scale; empirically, RLAU and CLAU have virtually identical params, FLOPs, memory, and runtime. Thus, the systematic accuracy gains of CLAU are achieved without any meaningful increase in computational footprint.

**Figure 6:**
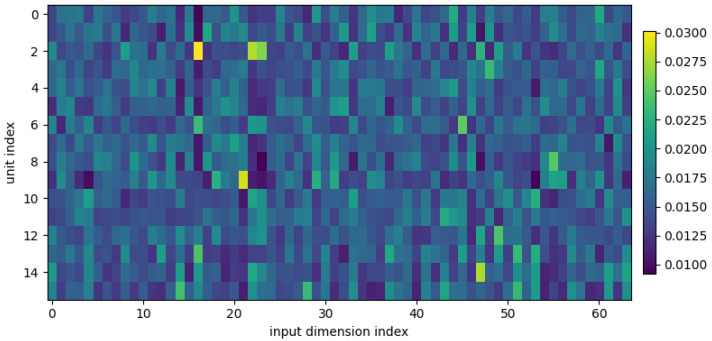
Heatmap of learned weights for the MNIST RLAU head.

Figure 6 visualizes the learned weights of the MNIST RLAU head. Each column corresponds to one of the 640 backbone features, and intensity encodes the learned importance. The weight pattern is clearly non-uniform, with pronounced concentration on a small subset of feature directions. This provides a direct interpretability artifact: the head computes an explicit normalized attribution distribution over the backbone representation. In contrast to pointwise activations (e.g., ReLU), which do not expose an explicit mechanism for global feature attribution, an LAU head yields a structured importance profile that can be inspected post hoc and compared across datasets or training runs.

Across all datasets, CLAU achieves consistently higher accuracy than RLAU while remaining computationally indistinguishable in this backbone-dominated regime. Since the only architectural difference is the aggregation operator in the head, the improvement can be attributed to the enlarged functional family induced by the complex moment (phase modulation through *b*) and the real-valued decoder (α,β,γ,δ), which enables richer nonlinear readouts of the complex aggregate. The experiments therefore highlight a central practical advantage of CLAU: it yields systematic gains for free in large-scale regimes where the incremental cost of the head is negligible.

Taken together, the image-based results establish that Lehmer-based activations provide strong predictive performance, intrinsic interpretability, and negligible overhead when deployed as compact decision-stage aggregators. Even with a single aggregation-based neuron in the head, LAU models remain competitive with modern deep classifiers, supporting the view that aggregation-based nonlinearities are a principled and efficient alternative to conventional pointwise activations.

## 6. Conclusions and Future Research

This work introduced Lehmer Activation Units (LAUs) as a principled alternative to conventional pointwise nonlinearities in neural networks. By grounding the activation function in the Lehmer transform, LAUs unify adaptive feature weighting and nonlinear aggregation within a single differentiable operator. Both real-valued and complex-valued formulations were developed, enabling magnitude-driven aggregation as well as phase-sensitive interactions. The resulting activation units are intrinsically interpretable, as their parameters directly encode feature importance and aggregation behavior, and are fully compatible with standard optimization procedures and architectural components.

Empirical results on tabular and image-based benchmarks demonstrate that LAU-based networks can achieve strong predictive performance using remarkably compact architectures. In particular, extremely shallow models with only a single Lehmer unit were sufficient for competitive tabular classification, while deep convolutional networks equipped with a single LAU-based decision-stage aggregator remained effective across diverse image datasets. These findings highlight that aggregation-based activations can concentrate expressive power within individual neurons, reducing architectural complexity while preserving performance and improving transparency.

Several directions for future research naturally follow from this work. Beyond classification, LAUs are well suited to regression problems where adaptive aggregation of heterogeneous features is essential. The complex-valued formulation is particularly promising for time-series and signal processing tasks involving oscillatory, spectral, or phase-dependent structure, such as biomedical signals, communications data, and financial time series. More broadly, the interpretability and aggregation-based inductive bias of LAUs make them appealing for scientific machine learning and physics-informed modeling, where preserving structural relationships among variables is critical. In addition, systematic comparisons between LAU-based activations and architectural aggregation mechanisms such as attention modules, dynamic convolution, or self-explaining networks constitute an important direction for future work. Further theoretical analysis of expressivity, approximation rates, optimization behavior, and generalization properties of Lehmer-based architectures also remains an important avenue for future work.

Overall, this work demonstrates that carefully designed aggregation-based activation functions can serve as a powerful foundation for building compact, interpretable, and expressive neural models, and suggests a fruitful direction for future research at the intersection of mathematical analysis and modern machine learning.

## Figures and Tables

**Table 1:** Performance of Lehmer Activation Units on tabular datasets (mean accuracy with standard deviation) under stratified 10-fold cross-validation with 20 repeats.

Activation	Iris	Wine	WBC
ReLU	58.60% (30.28%)	65.64% (27.96%)	78.33% (17.96%)
ELU	94.63% (11.06%)	93.71% (11.58%)	96.29% (9.00%)
GELU	91.87% (12.39%)	89.31% (14.16%)	80.24% (18.14%)
Swish	96.50% (6.86%)	95.70% (9.05%)	90.30% (15.03%)
Mish	96.23% (9.39%)	96.40% (8.67%)	90.88% (14.48%)
RLAU	98.93% (2.54%)	97.89% (3.32%)	98.74% (1.50%)
CLAU	98.60% (4.26%)	97.82% (5.61%)	98.80% (1.46%)

**Table 2:** Performance summaries of Lehmer Activation Units (parentheses show ratios relative to ReLU).

Dataset	Params			FLOPs			Time (s)		
	ReLU	RLAU	CLAU	ReLU	RLAU	CLAU	ReLU	RLAU	CLAU
Iris	32	32	37	59	74 (1.25)	156 (2.64)	0.42	0.77 (1.85)	1.24 (2.98)
Wine	203	203	208	410	443 (1.08)	615 (1.50)	0.49	0.92 (1.89)	1.52 (3.11)
WBC	1926	1926	1931	3905	4032 (1.03)	4674 (1.20)	1.43	2.50 (1.75)	3.92 (2.74)

**Table 3:** Architecture used for image classification experiments.

Component	Specification
Backbone	WideResNet-28-10
Feature dimension	640 (fixed across datasets)
Normalization	BatchNorm (backbone), LayerNorm (pre-head)
Head	Single RLAU/CLAU unit (640 → 1)
Residual head	Projection to feature space + residual injection
Classifier	Linear layer on enhanced features
Loss	Cross-entropy

**Table 4:** Dataset-specific augmentation policies used for image experiments.

Dataset	Augmentation Policy
MNIST	Random crop (pad = 4), random rotation (10°), RandAugment (1,6)
EMNIST	Random crop (pad = 4), random rotation (10°), RandAugment (1,6)
KMNIST	Random crop (pad = 4), random rotation (10°), RandAugment (1,6)
FMNIST	Random crop (pad = 4), random rotation (10°), RandAugment (1,6)
SVHN	Random crop (pad = 4), RandAugment (1,8), random erasing (p=0.1)
CIFAR-10	Random crop, horizontal flip, RandAugment (2,12), MixUp, CutMix
CIFAR-100	Random crop, horizontal flip, RandAugment (2,14), MixUp, CutMix
Tiny-ImageNet	Random resized crop, horizontal flip, color jitter, RandAugment, MixUp, CutMix

**Table 5:** Classification accuracy of Lehmer Activation Units on image benchmarks.

Dataset	RLAU	CLAU
MNIST	99.67%	99.72%
EMNIST	98.73%	98.83%
KMNIST	98.43%	98.53%
FMNIST	93.39%	93.89%
SVHN	95.87%	95.98%
CIFAR-10	97.25%	97.66%
CIFAR-100	83.16%	83.83%
Tiny-ImageNet	64.51%	65.06%

**Table 6:** Computational efficiency and resource usage of LAU-based models.

Dataset	Activation	Params (M)	FLOPs (G)	GPU Mem (GB)	Time/Epoch (s)
MNIST	RLAU	36.48	5.248	3.07	20.85
	CLAU	36.48	5.248	3.07	20.80
EMNIST	RLAU	36.51	5.248	3.07	39.63
	CLAU	36.51	5.248	2.92	39.06
KMNIST	RLAU	36.48	5.248	3.08	20.97
	CLAU	36.48	5.248	3.08	20.82
FMNIST	RLAU	36.48	5.248	3.08	21.05
	CLAU	36.48	5.248	3.08	20.86
SVHN	RLAU	36.48	5.248	3.79	23.67
	CLAU	36.48	5.248	3.80	22.44
CIFAR-10	RLAU	36.48	5.248	3.80	19.62
	CLAU	36.48	5.248	2.90	18.87
CIFAR-100	RLAU	36.54	5.248	2.90	18.88
	CLAU	36.54	5.248	2.91	19.00
Tiny-ImageNet	RLAU	36.60	20.992	10.62	95.67
	CLAU	36.60	20.992	10.62	95.89

## Data Availability

All data and code that support the findings of this study are openly available at: https://github.com/chimera1001/Lehmer_Neural_Network.git (accessed on 29 January 2026).
